# Rootstock Alleviates Salt Stress in Grafted Mulberry Seedlings: Physiological and PSII Function Responses

**DOI:** 10.3389/fpls.2018.01806

**Published:** 2018-12-11

**Authors:** Huihui Zhang, Xin Li, Shubo Zhang, Zepeng Yin, Wenxu Zhu, Jinbo Li, Liang Meng, Haixui Zhong, Nan Xu, Yining Wu, Guang yu Sun

**Affiliations:** ^1^College of Resources and Environment, Northeast Agricultural University, Harbin, China; ^2^College of Life Sciences, Northeast Forestry University, Harbin, China; ^3^College of Forestry, Shenyang Agricultural University, Shenyang, China; ^4^Natural Resources and Ecology Institute, Heilongjiang Academy of Sciences, Harbin, China; ^5^Hubei Wel-Safe Biotechnology Co., Ltd., Wuhan, China

**Keywords:** grafted mulberry, salt stress, ion absorption, chlorophyll fluorescence, reactive oxygen species (ROS)

## Abstract

This study investigated the effect of NaCl stress on Na^+^ and K^+^ absorption and transport by roots, nitrogen and phosphorus content in leaves, PSII photochemical activity and reactive oxygen species (ROS) in leaves of mulberry own-root seedlings and grafted seedlings. To determine the response, own-root seedlings of a high yielding mulberry cultivar, Tieba mulberry (*Morus alba* L.), and the grafted seedlings, obtained by using Qinglong mulberry with high salt tolerance as rootstock and Tieba mulberry as scion, were used. The Na^+^ content in roots and leaves of grafted seedlings was significantly lower than that in own-root seedlings under salt stress; while K^+^ content in roots and leaves of grafted seedlings was significantly higher than that in own-root seedlings. The root activity in grafted seedlings was significantly higher than that in own-root seedlings, as well as the content of nitrogen, phosphorous and water. PSII photochemical activity in leaves of grafted seedlings was less significantly affected by salt stress compared to own-root seedlings. The electron transport at the acceptor side of PSII from *Q*_A_ to *Q*_B_ was less affected by salt stress, which resulted in a significantly lower ROS content in leaves of grafted seedlings than that of own-root seedlings. Therefore, grafting high-yielding and good-quality Tieba mulberry with salt tolerant Qinglong mulberry as rootstock showed a relatively high salt tolerance. This may be because (1) the root system of rootstock presented high Na^+^ resistance and has selective absorption capacity for Na^+^ and K^+^ (2) the root system of rootstock prevented excess Na^+^ from being transported to aerial parts in order to reduce adverse effects of Na^+^ (3) the root system of rootstock had enhanced root activity under salt stress, which accelerated water and nutrient absorption (4) the leaves of grafted seedlings had higher PSII photochemical activity and electron transport rate compared with those of own-root seedlings under salt stress, which effectively reduced ROS burst mediated by photosynthesis and reduced oxidative damage.

## Introduction

According to the statistics, 20% of arable area in the whole world is affected by soil salinization (Zhu, 2001). Due to industrial pollution and over-fertilization, the secondary salinized area is continuously expanding, which severely threatens crop security and ecosystem stability (Landi et al., 2017). Salts in the soil are indispensable for normal growth of plants (Wang et al., 2001). But excess salts cause osmotic stress (Munns et al., 2006) and ion toxicity (Ali et al., 2017), and interfere with soil nutrient balance (Yan et al., 2006) affecting plant growth and physiological functions. Salt stress affects chlorophyll synthesis and photosynthetic ability of plants (Gong et al., 2013; Dąbrowski et al., 2016), which often leads to reduction in PSII activity, inhibition of electron transport, limitation to carbon assimilation, and peroxidation or dissociation of thylakoid membrane (Mitsuya et al., 2000; Takahashi et al., 2017).

In the long-term, plants have evolved mechanisms of salt tolerance such as exclusion of salts (Zhao et al., 2016a), accumulation of salts for osmotic adjustment (Moghaieb et al., 2004; Song et al., 2006) and activation of anti-oxidative system (Askari et al., 2006; Wu et al., 2012) by the roots. The plant root system also controls passing in and out of salts using ion channels (NSCC, voltage dependent type non-selective cation channel and GLR, glutamate activation channel) (Kav et al., 2004; Jiang et al., 2007; Cheng et al., 2009) or transporters (Na^+^/H^+^ antiporter and HKT, high affinity K^+^ transporter), and/or adapt to salt stress by adjusting intracellular ion equilibrium via ion compartmentation (Apse and Blumwald, 2007). The mechanisms of adaptation by roots to salt stress are different in different species or varieties. Therefore, utilizing grafting technique to select a salt tolerant root system as rootstock may increase salt tolerance in plants. In grafting, the root system of rootstock replaces that of the scion. In this process, if the rootstock is effectively selected, it will strengthen nutrient and water absorption (Kumar et al., 2018), increase carbon and nitrogen metabolism (Shahid et al., 2018), and further increase salt tolerance in plants. Grafting technique not only maintains good strains of seeds and fix heterosis, but also increases the resistance of plants (Nogueira Filho et al., 2010; Penella et al., 2014; Balal et al., 2017). Grafting can also increase the salt tolerance in plants (Nastou et al., 2002; Aboutalebi, 2009). Grafting in tomato reduced Na^+^ and Cl^-^ transport by roots to aerial parts under salt stress (Estañ et al., 2005) and accelerated K^+^ absorption by roots to adapt to salt stress (Fan et al., 2011). It increased photosynthetic ability, and increased salt tolerance by increasing the activity of antioxidant enzymes (He et al., 2009). The degree of photoinhibition in grafted cucumber leaves was significantly relieved under salt stress (Huang et al., 2011; Liu et al., 2012), and the nitrogen metabolism ability was strengthened (Liu et al., 2013). Grafted seedlings absorbed more nitrogen and phosphorous under salt stress (Uygur and Yetisir, 2009).

Mulberry is one of the major economic tree species which restores ecology and increases agriculture income in northern frigid and salinized areas of China (Li et al., 2016). Mulberry cultivar in local northern areas have high salt tolerance and low temperature tolerance, but the leaf yields are low and the quality is bad. Meanwhile, mulberry cultivar in the southern areas have high yield and good quality. Their adaption to adverse environmental conditions such as low temperature, drought and salinization is poor. Therefore, grafting in mulberry using cultivar with resistance ability of the northern areas as rootstock and cultivar with high yield and good quality of the southern areas as scion is one of the important approaches to aid extension of mulberry into northern alpine salinized area. We previously found that grafted mulberry had a relatively high salt tolerance, but the mechanisms behind tolerance need further investigation. Thus, the current study used southern high yield and good quality “Tieba mulberry” own-root seedlings as control, and compared with the grafted seedlings generated from “Qinglong mulberry,” the cultivar in the northern areas having low yield but high resistance, as rootstock to graft Tieba mulberry. The study investigated the response of Na^+^ and K^+^ content in roots and leaves, activity of roots, water, nitrogen and phosphorous content in leaves, PSII function under different concentrations of NaCl stress. The study analyzed the mechanisms behind salt tolerance in grafted mulberry seedlings in terms of ion absorption and photosynthesis, in order to provide a theoretical basis for reasonable extension of mulberry planting into salinized areas.

## Materials and Methods

### Experimental Materials and Treatments

The mulberry own-root seedlings and grafted seedlings were provided by Sericultural Research Institute, Heilongjiang. The experimental material, own-root seedlings and grafted seedlings rootstock are annual, which were grafted and survived in May, 2017; the scion and rootstock have completed healing. The experiment was carried out in the soil lab in Northeast Agricultural University in August, 2017. Mulberry own-root seedlings and grafted seedlings with relatively uniform growth were selected, pulled out of culture substrate, and were grown in 1/2 Hoagland complete solution. The Hoagland complete solution consists of 0.75 × 10^-3^ mol L^-1^ K_2_SO_4_, 0.65 × 10^-3^ mol L^-1^ MgSO_4_, 0.1 × 10^-3^ mol L^-1^ KCl, 2.0 × 10^-3^ mol L^-1^ Ca(NO3)_2_, 0.25 × 10^-3^ mol L^-1^ KH_2_PO_4_, 1.0 × 10^-5^ mol L^-1^ H_3_BO_3_, 1 × 10^-6^ mol L^-1^ MnSO_4_, 1 × 10^-7^ mol L^-1^ CuSO_4_, 1 × 10^-6^ mol L^-1^ ZnSO_4_, 5 × 10^-9^ mol L^-1^ (NH_4_)_6_Mo_2_O_4_, and 1.0 × 10^-4^ mol L^-1^ Fe-EDTA (pH of the solution was adjusted to around 7.0 using KOH or H_2_SO_4_). The seedlings were cultivated for 30 days. Aeration was provided using booster pump on a daily basis and the solution was changed every 5 days. Mulberry own-root seedlings and grafted seedlings were grown in 1/2 Hoagland complete solution with 0(CK), 50, 100, 150, and 200 mmol L^-1^ of NaCl to induce salt stress. Five strains of own-root seedlings and five strains of grafted seedlings were maintained as replicates for each salt concentration. Seedlings were analyzed for different physiological parameters after growing for 7 days.

### Measurement of Parameters

Chlorophyll a fluorescence transient (OJIP curve) was measured in the second (from top to bottom) fully expanded leaf using Handy-PEA chlorophyll fluorometer (Hansatech Instruments, United Kingdom). Leaves were dark-adapted for 30 min, and each measurement was repeated five times. The OJIP curve was assessed under 3000 μmol ⋅ m^-2^ ⋅ s^-1^ pulse red light, and the recording of the fluorescence signals started at 10 μs after exposure and stopped at 1 s after exposure. The O, J, I, and P points on OJIP curve correspond to the time points 0, 20, 30, and 1000 ms, respectively. The relative fluorescence of O point was defined as 0 and of P point as 1. The OJIP curve was standardized using the equations *V*_O-P_ = (*F*_t_-*F*_o_)/(*F*_m_-*F*_o_) and *V*_O-J_ = (*F*_t_-*F*_o_)/(*F*_J_-*F*_o_). The relative variable fluorescence *V*_J_ and *V*_K_ of J point at 2 ms on *V*_O-P_ curve and K point at 0.3 ms on *V*_O-J_ curve were obtained. A JIP-test analysis was conducted on the OJIP curve to obtain the maximum photochemical efficiency of PSII (*F*_v_/*F*_m_) and the photosynthetic performance index based on the absorbed light energy (*PI*_ABS_). The JIP-test method by Strasser et al. (1995). Measured part of the leaf was selected from an area between the 3rd and the 4th leaf vein from bottom, with a distance of approximately 2 cm to the major leaf vein.

The content of superoxide radical (O_2-_) release was determined according to Zhang et al. (2007) based on the principles that superoxide radicals react with hydroxylamine hydrochloride to from nitrite, nitrite reacts with *p*-aminobenzene sulfonamide and naphthylamine, and form nitrite a pink colored complex. The reaction mixture contained 50 nM phosphate buffer (PH 7.8), 1.0 mM hydroxylamine hydrochloride and 100 μL supernatant. The cuvette was incubated at 30°C for 30 min, and 17 mM *p*-aminobenzene sulfonamide and 7 mM 1-naphthylamine were added and kept in 30°C warm water bath for another 15 min. The absorbance at 530 nm was recorded and the amount of ${\text{O}}_{2}^{•-}$ release was calculated based on a standard nitrite curve (rang from 0 to 2 ${\text{NaNO}}_{2}^{•}$). H_2_O_2_ content was measured using the method of Alexieva et al. (2001). The reaction mixture consisted 0.5 mL 0.1% trichloroacetic acid (TCA) leaf extract supernatant, 0.5 mL of 100 mM K-phosphate buffer and 2 mL reagent (1 M KI w/v in fresh double-distilled water H_2_O). The blank probe consisted of 0.1% TCA in the absence of leaf extract. The reaction was developed for 1 h in darkness and absorbance measured at 390 nm. The amount of hydrogen peroxide was calculated using a standard curve prepared with known concentrations of H_2_O_2_. Root activity was measured using 2,3,5-Triphenyte-trazoliumchloride (TTC) method (Zhao et al., 1998). A 0.5 g root tip sample was weighed and fully immersed in a small beaker with a solution of 5 mL TTC (0.4%) and 5mL phosphate buffer (pH 7.0). After the sample was kept in dark conditions at 37°C for 2 h, 2 mL of sulfuric acid (1 mol L^-1^) was immediately added to stop the reaction. The treated root tip sample was then dried with filter paper and ground thoroughly with a mortar using 3–4 mL ethyl acetate. Its residue was washed 2–3 times with a small amount of ethyl acetate. All solutions were moved to a single calibration tube and the total amount was adjusted to 10 mL by adding ethyl acetate. Finally, colorimetric analysis was conducted by spectrophotometer at a wavelength of 485 nm, and the TTC reduction amount was calculated by standard curve, i.e., root activity. All measurements were repeated three times (biological experiments).

After measuring chlorophyll fluorescence and different physiological parameters, roots and leaves of the seedlings were harvested and dried at 80°C. Water content of the leaves was calculated using the equation; water content = [(fresh weight – dry weight) / dry weight] × 100%. For salt and nutrient analysis in leaves and roots, they were dried, ground and sieved though a 40-mesh sieve. Measurement of potassium and sodium content in roots and leaves was done by heating, and digestion using concentrated H_2_SO_4_-H_2_O_2_, and measured using flame photometry. Micro-Kjeldahl method and UV-visible spectrophotometry method were used for the measurement of leaf nitrogen and phosphorous content, respectively (Bao, 2005). All measurements were repeated three times (biological experiments).

### Data Process and Statistical Methods

Microsoft Excel and SPSS software were used for statistical analysis. Data in graphs are mean ± standard error (SE). One-way Analysis of Variance (One-way ANOVA) and Least Significant Difference (LSD) method were adopted to compare the means of different groups.

## Results and Analysis

### Na^+^ and K^+^ Content in Roots and Leaves of Mulberry Own-Root Seedlings and Grafted Seedlings Under Salt Stress

**FIGURE 1:**
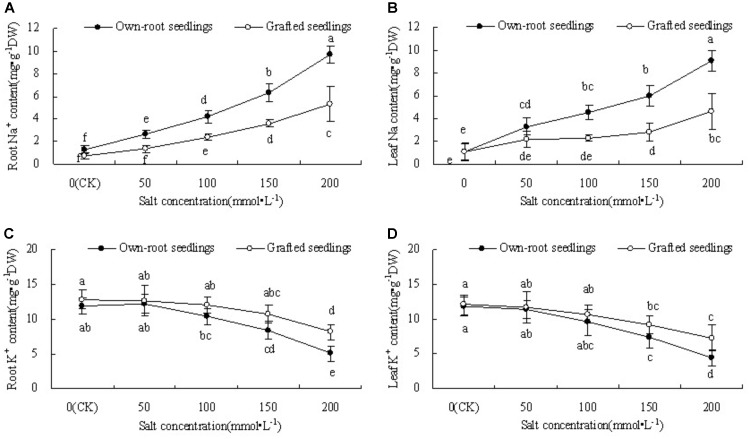
Root and leaf Na^+^ content **(A,B)** and K^+^ content **(C,D)** of mulberry own-root seedlings and grafted seedlings under salt stress.

Figures 1A,B show that the root Na^+^ content of mulberry own-root seedlings and grafted seedlings significantly increased with increase in salt concentration. The root Na^+^ content of own-root seedlings was significantly higher than that of grafted seedlings at each salt concentration, and the difference between the two seedling types increased with increase in salt concentration. Similar to root Na^+^ content, the leaf Na^+^ content of mulberry own-root seedlings and grafted seedlings demonstrated a significant increase with increase in salt concentration. The leaf Na^+^ content of own-root seedlings showed an overall linear increase with increase in salt concentration. However, the leaf Na^+^ content of grafted seedlings showed smaller increase at salt concentrations lower than 150 mmol L^-1^, which further increased as the salt concentration increased to 200 mmol L^-1^.

Figures 1C,D show that the root and leaf K^+^ content of mulberry own-root seedlings and grafted seedlings decreased with increase in salt concentration. However, with increase in salt concentration, the root and leaf K^+^ content of grafted seedlings became gradually higher than that of own-root seedlings. The difference in both root and leaf K^+^ content between the two seedling types was insignificant at salt concentrations lower than 150 mmol ⋅ L^-1^. At salt concentration 200 mmol L^-1^, grafted seedlings showed 50.78% (*P* < 0.05) and 55.91% (*P* < 0.05) higher root and leaf K^+^ content than those of own-root seedlings, respectively.

### Root Vigor and Leaf Water Content of Mulberry Own-Root Seedlings and Grafted Seedlings Under Salt Stress

**FIGURE 2:**
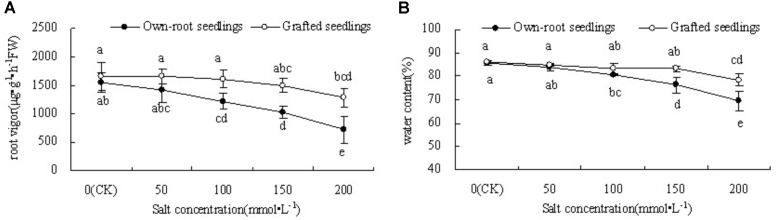
Root vigor **(A)** and leaf water content **(B)** of mulberry own-root seedlings and grafted seedlings under salt stress.

Figure 2A shows that when the salt concentration was 0, the root vigor of mulberry grafted seedlings was slightly higher than that of own-root seedlings, but the difference was insignificant. With increase in salt concentration, there was a decrease in root vigor in both seedling types; the decrease was significantly more in own-root seedlings compared to grafted seedlings. The grafted seedlings showed a significant decrease in root vigor under salt stress only at salt concentration 200 mmol L^-1^.

Figure 2B shows that at a salt concentration of 50 mmol L^-1^, the leaf water content of both own-root seedlings and grafted seedlings had no significant difference compared with CK. As the salt concentration increased to 100 mmol L^-1^, the leaf water content of own-root seedlings started to decrease significantly, which further decreased with increase in salt concentration. However, with increase in salt concentration, the leaf water content of grafted seedlings showed no significant change compared with CK, except for the significant decrease at a higher salt concentration of 200 mmol L^-1^. The leaf water content of grafted seedlings was significantly lower than that of own-root seedlings when the salt concentration was 150 and 200 mmol ⋅ L^-1^.

### Leaf N and P Content of Mulberry Own-Root Seedlings and Grafted Seedlings Under Salt Stress

**FIGURE 3:**
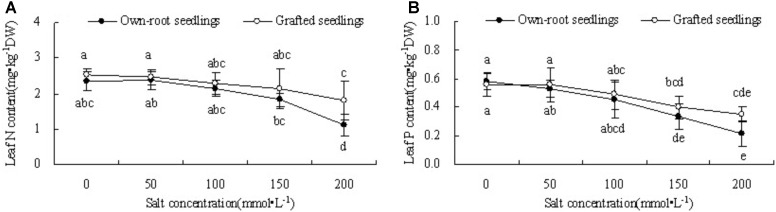
Leaf N content **(A)** and P content **(B)** of mulberry own-root seedlings and grafted seedlings under salt stress.

Figure 3A shows that with increase in salt concentration, the leaf N content of mulberry own-root seedlings and grafted seedlings decreased. The leaf N content of own-root seedlings and grafted seedlings at salt concentrations lower than 150 mmol L^-1^ was not significantly different from CK; although the leaf N content of grafted seedlings was always slightly higher than that of own-root seedlings, the difference was insignificant. As the salt concentration increased to 200 mmol L^-1^, the leaf N content of both own-root seedlings and grafted seedlings significantly decreased; and the leaf N content of grafted seedlings was 38.33% (*P* < 0.05) higher than that of own-root seedlings. As shown in Figure 3B, the leaf P content of own-root seedlings and grafted seedlings decreased with increase in salt concentration. The leaf P content of both own-root seedlings and grafted seedlings had no significant change at a salt concentration lower than 100 mmol L^-1^, and the difference between the two seedling types was not significant. As the salt concentration increased to 150 and 200 mmol L^-1^, the leaf P content of both own-root seedlings and grafted seedlings significantly decreased. The leaf P content of grafted seedlings was 16.32% (*P* > 0.05) and 38.44% (*P* > 0.05) higher than that of own-root seedlings, though the difference was not significant.

### Leaf Chlorophyll Fluorescence Parameters of Mulberry Own-Root Seedlings and Grafted Seedlings Under Salt Stress

#### OJIP Curve

**FIGURE 4:**
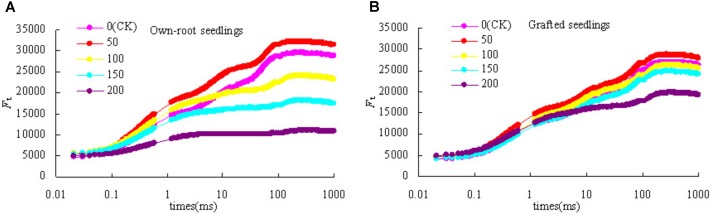
OJIP curve of mulberry own-root seedlings **(A)** and grafted seedlings **(B)** under salt stress.

Figure 4 shows that with increase in salt concentration, OJIP curve of own-root seedlings and grafted seedlings both obviously changed. The relative fluorescence intensity at point O of own-root seedlings and grafted seedlings less changed at different salt concentrations. However, the relative fluorescence intensities at points J, I, and P decreased to different degrees, especially the relative fluorescence intensity at point P showed the largest decreasing magnitude. The difference in OJIP curve of grafted seedlings was significantly lower compared to own-root seedlings under different salt concentrations, and the relative fluorescence intensities at points I and P significantly decreased only at a salt concentration of 200 mmol L^-1^.

#### PSII Photochemical Activity

**FIGURE 5:**
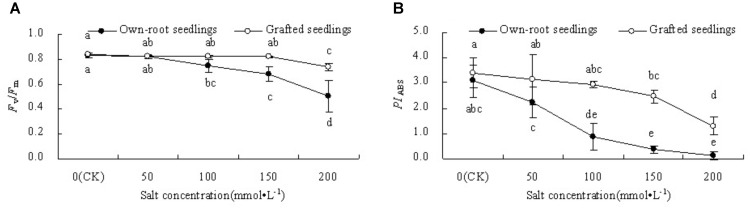
Leaf *F*_v_/*F*_m_ **(A)** and *PI*_ABS_ **(B)** of mulberry own-root seedlings and grafted seedlings under salt stress.

Figure 5A shows that *F*_v_/*F*_m_ in leaves of mulberry grafted seedlings did not change at salt concentrations lower than 150 mmol L^-1^, while it decreased 12.32% (*P* < 0.05) compared to CK at salt concentration 200 mmol L^-1^. The *F*_v_/*F*_m_ of own-root seedlings decreased significantly compared with CK at salt concentrations above 50 mmol L^-1^. The *F*_v_/*F*_m_ of grafted seedlings at 150 and 200 mmol L^-1^ were significantly higher than that of own-root seedlings. As shown in Figure 5B, *PI*_ABS_ in leaves of both own-root seedlings and grafted seedlings decreased with increase in salt concentration. *PI*_ABS_ of own-root seedlings decreased significantly compared with CK when the salt concentration was increased to 100 mmol L^-1^, while *PI*_ABS_ of grafted seedlings decreased significantly compared with CK only when the salt concentration was increased to 150 mmol L^-1^. *PI*_ABS_ of grafted seedlings was significantly higher than that of own-root seedlings at all salt concentrations.

#### Electron Transfer Ability of PSII Donor and Acceptor

**FIGURE 6:**
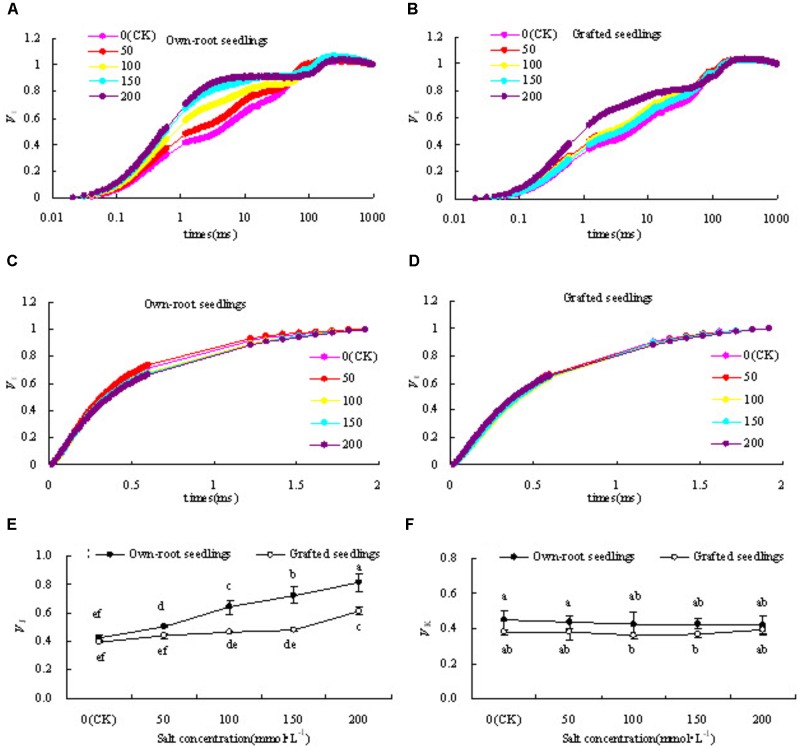
*V*_O-P_**(A,B)**, *V*_O-J_**(C,D)**, *V*_J_ **(E)** and *V*_K_**(F)** of mulberry own-root seedlings and grafted seedlings leaf under salt stress.

Figures 6A,B show that the relative variable fluorescence (*V*_J_) at point J (2 ms) on the standardized O-P curve in leaves of mulberry own-root seedlings and grafted seedlings under salt stress increased to different degrees compared with CK. With increase in salt concentration, *V*_J_ of own-root seedlings increased, and reached a level significantly different from CK at different salt concentrations. However, the increasing magnitude of *V*_J_ of grafted seedlings was significantly lower than that of own-root seedlings, and at salt concentrations lower than 150 mmol L^-1^. Figure 6E shows no significant difference in *V*_J_ of own-root seedlings when compared to CK.

Figures 6C,D shows that the variation magnitudes of standardized O-J curve in leaves of own-root seedlings and grafted seedlings both were small compared with CK, especially that of grafted seedlings basically had no obvious change. In Figure 6F the quantitative analysis shows that *V*_K_ in leaves of own-root seedlings at different salt concentrations was slightly higher than that of grafted seedlings, with the difference being insignificant. The *V*_K_ in leaves of own-root seedlings and grafted seedlings did not change with change in salt concentration.

### Leaf ROS Content and Membrane Lipid Peroxidation

**FIGURE 7:**
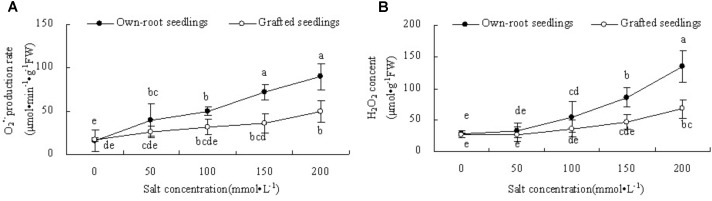
The ${\text{O}}_{2}^{•-}$ production rate **(A)** and H_2_O_2_ content **(B)** in the leaves of mulberry own-root seedlings and grafted seedlings under salt stress.

Figure 7A shows that with increase in salt concentration, the ${\text{O}}_{2}^{•-}$ production rate in the leaves of own-root seedlings significantly increased, while the ${\text{O}}_{2}^{•-}$ production rate in the leaves of grafted seedlings had no significant difference compared to CK at salt concentrations lower than 150 mmol L^-1^. At salt concentration 200 mmol L^-1^, the ${\text{O}}_{2}^{•-}$ production rate in leaves of own-root seedlings significantly increased compared with CK. The ${\text{O}}_{2}^{•-}$ production rate in leaves of grafted seedlings was lower than that of own-root seedlings at different salt concentrations, and the difference increased with increase in salt concentration. In Figure 7B it is shown that at a salt concentration of 50 mmol L^-1^, the leaf H_2_O_2_ content of own-root seedlings and grafted seedlings had no significant difference compared with CK, and these showed no significant difference. However, with increase in salt concentration, the leaf H_2_O_2_ content of own-root seedlings sharply increased, which was significantly higher than that of CK at a salt concentration of 100 mmol L^-1^ and above. The magnitude of increase in leaf H_2_O_2_ content of grafted seedlings was obviously lower than that of own-root seedlings.

## Discussion

### Grafting in Mulberry Reduces Na^+^ Absorption by Roots and Transfer to Leaves Under Salt Stress

Plants have evolved a series of salt tolerance mechanisms to adapt to salt stress. These include selective absorption of Na^+^ and K^+^ (Peng and Tang, 2004; Zhang et al., 2007*;* Song et al., 2009; Wang et al., 2009); regionalize salts entering the body at organ, tissue, and cellular levels (e.g., reserving Na^+^ in the root system to prevent it from getting transferred to aerial parts); transfer of ions to the vacuole via Na^+^/H^+^ antiporter, H^+^-ATPase and H^+^-PPase (Wang et al., 2001; Qiu et al., 2007; Flowers and Colmer, 2008; Chen et al., 2010). In the current study in mulberry, root and leaf Na^+^ content of own-root seedlings and grafted seedlings increased with increase in salt concentration. The root and leaf Na^+^ content of grafted seedlings were significantly lower than those of own-root seedlings, indicating that roots of grafted seedlings had relatively high salt exclusion ability under salt stress and lowered Na^+^ toxicity due to reduced absorption. Although root Na^+^ content of grafted seedlings demonstrated a significant increase with increase in salt concentration, the leaf Na^+^ content showed smaller increase at salt concentrations below 150 mmol L^-1^, Taken collectively, this suggest that roots of grafted seedlings reduce transfer of Na^+^ to leaves and reduce effect of Na^+^ on aerial parts. Plant damage triggered by salt stress is related to toxicity effect of Na^+^ and osmotic stress by larger ions (Munns et al., 2006). To adapt to osmotic stress, plants increase the water absorbing ability of roots by absorbing a large amount of K^+^, Ca^2+^, and Mg^2+^ (Khan et al., 2000; Yang et al., 2008), and synthesizing soluble small organic molecular matters as osmotic regulators (Moghaieb et al., 2004; Duan et al., 2005). In this experiment, although the root and leaf K^+^ content of own-root seedlings and grafted seedlings had no significant difference at low salt concentration, the root and leaf K^+^ content of grafted seedlings showed a significant increase with increase in salt concentration compared to own-root seedlings. This indicates that grafted seedlings increase K^+^/Na^+^ ratio to reduce toxicity effect of Na^+^ by selective absorption of Na^+^ and K^+^ at high salt concentration. Therefore, mulberry grafted seedlings mitigate toxicity of Na^+^ through salt exclusion effect, by preventing transfer of Na^+^ to aerial parts, and by maintaining cellular osmotic equilibrium by selectively absorbing more K^+^.

### Grafting in Mulberry Relieves Damage During Salt Stress in the Roots and Promotes Absorption of Nutrients Such as N, P, K, and Water

Root system is the part of a plant that is directly stimulated by soil salts (Zhao et al., 2016b). It is the major organ that absorbs nutrients and water, and helps maintain morphological characteristics and vigor under adverse conditions (Giehl et al., 2014; Kellermeier et al., 2014). The morphology and function of plant root system are essential for salt tolerance ability (Srinivasrao et al., 2004; Galvan-Ampudia and Testerink, 2011). The current study revealed that the root vigor of mulberry grafted seedlings was moderately higher than that of own-root seedlings under salt stress. With increase in salt concentration, the decrease in root vigor of mulberry grafted seedlings was significantly lower than that of own-root seedlings. This indicates that the rootstock of the grafted seedlings had relatively high root vigor and improved tolerance to salt stress. Plant root system is the major organ absorbing water and nutrients, and some studies found that grafting could promote the absorption of the plant to nutrients and water (Uygur and Yetisir, 2009). In this experiment, root vigor, leaf water content and N-P-K content of mulberry grafted seedlings under salt stress were higher than those of own-root seedlings, which showed significant difference at higher salt concentration. Here, the mulberry grafted seedlings demonstrated a relatively high root vigor under salt stress, facilitating better absorption of water and nutrients. Thus the current study provides basis for improving both vigor and tolerance of mulberry grafted seedlings under salt stress.

### Grafting in Mulberry Relieves PSII Photoinhibition Under Salt Stress and Reduces ROS Damage

PSII reaction centre is sensitive to salt stress, and studies have proven that salt stress inhibits PSII photochemical activity (Lu and Zhang, 2000). *F*_v_/*F*_m_ and *PI*_ABS_ are important indicators of PSII photochemical activity, and the sensitivity of *PI*_ABS_ is larger than that of *F*_v_/*F*_m_ (Zhang et al., 2017, 2018a). In this experiment, with increase in salt concentration, the decrease in *F*_v_/*F*_m_ and *PI*_ABS_ of mulberry grafted seedlings were significantly lower than that of own-root seedlings. This indicates that salt stress inhibits PSII photochemical activity of own-root seedlings and grafted seedlings. The degree of photoinhibition in grafted seedlings was relieved, which was similar to the results of Huang et al. (2011). In their study, grafting in cucumber resulted in increased photochemical activity. Chlorophyll fluorescence induction curve contains information on PSII donor and acceptor activity. The increase in relative variable fluorescence (*V*_K_) value at point K (0.3 ms) is considered as the specific marker for inhibition of activity at PSII electron donor side, especially the activity of oxygen evolution complex (OEC) (Jiang et al., 2008; Zhang et al., 2012). The increase in relative variable fluorescence (*V*_J_) at point J (2 ms) indicates that the electron transfer from *Q*_A_ to *Q*_B_ is blocked and there is accumulation of reduced *Q*_A_ (Haldimann and Strasser, 1999, Zhang A.Q. et al., 2016; Zhang H.H. et al., 2016). In this experiment, with increase in salt concentration, variation in *V*_K_ of both mulberry own-root seedlings and grafted seedlings was small. However, *V*_J_ sharply increased, indicating that the damage caused by salt stress to PSII activity mainly occurred at PSII acceptor side, and its effects on PSII donor side was relatively small. Some studies found that salt stress leads to up-regulation of OEC protein expression (Pang et al., 2010; Zhang et al., 2012), and that OEC is not sensitive to salt stress and a significant decrease in activity is also reported (Allakhverdiev et al., 2001; Abbasi and Komatsu, 2004; Park et al., 2004). However, when the damage to the PSII acceptor side was significantly larger than that to the donor side, it did not lead to increase in *V*_K_ (Zhang et al., 2018b). In the current study, *V*_K_ of mulberry own-root seedlings and grafted seedlings showed no significant change. This may be probably because salt stress did not directly cause damage to OEC or caused relatively larger damage to PSII acceptor side of own-root seedlings and grafted seedlings. Therefore, studies investigating OEC activity in mulberry seedlings under salt stress need to be conducted. The increasing magnitude of *V*_J_ in leaves of mulberry grafted seedlings under salt stress was significantly lower than that of the grafted seedlings, and *V*_J_ in leaves of grafted seedlings did not significantly at salt concentrations lower than 150 mmol L^-1^. The electron transfer at PSII acceptor side from *Q*_A_ to *Q*_B_ is blocked under salt stress, which was mainly associated with inhibition of D_1_ protein degradation or affect its turnover (Murata et al., 2007; Cheng et al., 2016). Therefore, the relatively lesser degree of inhibition in electron transfer at PSII acceptor side in mulberry grafted seedlings under salt stress might be related to lesser damage by salts to D_1_ protein.

**FIGURE 8:**
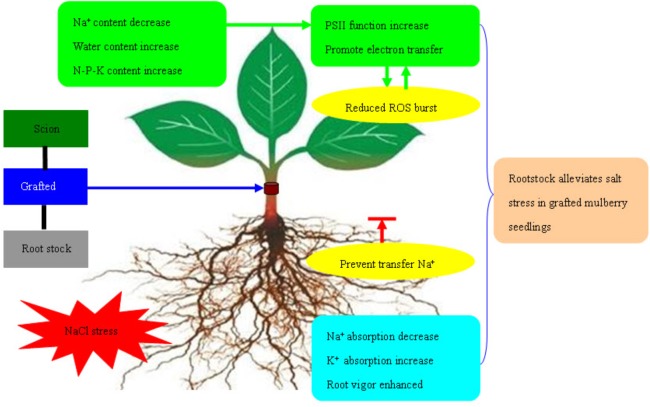
The mechanism of rootstock alleviates salt stress in grafted mulberry seedlings.

The transfer of electrons through molecules of PSII electron transport chain was blocked under adverse environmental conditions. The excess electron or light energy which is not utilized resulted in the production of ROS (Venkatesh et al., 2012; Long et al., 2017; Guo et al., 2018). Excess ROS damage the cells via serious oxidative effect (Chen et al., 2005). ROS mediated by photosynthesis first attacks the chloroplast membrane or inhibits synthesis of protein related to photosynthesis. This leads to reduction in PSII activity, induces production of more ROS, and forms a vicious cycle (Nishiyama et al., 2011). In the current experiment, with increase in salt concentration, the ${\text{O}}_{2}^{•-}$ production rate and H_2_O_2_ content in leaves of mulberry own-root seedlings and grafted seedlings increased. This was similar to the variation in *V*_J_, indicating that increased accumulation of ROS in leaves of own-root seedlings and grafted seedlings was directly related to the hindered electron transfer at PSII acceptor side. However, the ${\text{O}}_{2}^{•-}$ production rate and H_2_O_2_ content in the leaves of grafted seedlings were significantly lower than those of own-root seedlings. This proves that the oxidative damage due to salt stress in the leaves of mulberry grafted seedlings was significantly lower than that in own-root seedlings. Figure 8 shows that the mechanism of rootstock alleviates salt stress in grafted mulberry seedlings.

## Conclusion

Mulberry grafting using salt-tolerant rootstock reduced Na^+^ absorption by roots and transfer to leaves in grafted seedlings under salt stress. The leaves of grafted seedlings had relatively high root vigor under salt stress, promoting the absorption of nutrients such as N-P-K. Under salt stress, grafted seedlings had higher PSII photochemical activity compared to own-root seedlings. The inhibition of PSII electron transport under salt stress was relieved in grafted seedlings, thus effectively reducing the production of ROS. Therefore, grafting using Qinglong mulberry with salt tolerance as rootstock and Tieba mulberry with high yield and good quality as scion demonstrates improved salt tolerance.

## Author Contributions

NX, YW, XL, and HZ conceived and designed the experiments. NX and HZ wrote the manuscript and prepared the figures and/or tables. All the authors performed the experiments and analyzed the data.

## Conflict of Interest Statement

The authors declare that the research was conducted in the absence of any commercial or financial relationships that could be construed as a potential conflict of interest.
